# Targeted therapy strategies for melanoma brain metastasis

**DOI:** 10.1093/noajnl/vdab131

**Published:** 2021-11-27

**Authors:** Chantal Saberian, Paul Sperduto, Michael A Davies

**Affiliations:** 1 Department of Melanoma Medical Oncology, The University of Texas MD Anderson Cancer Center, Houston, Texas, USA; 2 Minneapolis Radiation Oncology, Minneapolis, Minnesota, USA

**Keywords:** brain metastasis, melanoma, targeted therapy

## Abstract

Melanoma is the most aggressive of the common forms of skin cancer. Metastasis to the central nervous system is one of the most common and deadly complications of this disease. Historically, melanoma patients with brain metastases had a median survival of less than 6 months. However, outcomes of melanoma patients have markedly improved over the last decade due to new therapeutic approaches, including immune and targeted therapies. Targeted therapies leverage the high rate of driver mutations in this disease, which result in the activation of multiple key signaling pathways. The RAS-RAF-MEK-ERK pathway is activated in the majority of cutaneous melanomas, most commonly by point mutations in the *Braf* serine-threonine kinase. While most early targeted therapy studies excluded melanoma patients with brain metastases, subsequent studies have shown that BRAF inhibitors, now generally given concurrently with MEK inhibitors, achieve high rates of tumor response and disease control in *Braf-*mutant melanoma brain metastases (MBMs). Unfortunately, the duration of these responses is generally relatively short- and shorter than is observed in extracranial metastases. This review will summarize current data regarding the safety and efficacy of targeted therapies for MBMs and discuss rational combinatorial strategies that may improve outcomes further.

Melanoma is the most aggressive of the common forms of skin cancer. One of the most common and devastating complications of this disease is the development of metastasis to the central nervous system (CNS), including the brain and the leptomeninges (LMD).^1^ Previous studies have reported that incidence of CNS metastasis among stage IV melanoma patients is as high as 50%, and CNS involvement was identified in approximately 80% of patients in an autopsy series.^2^ CNS involvement is detected in up to 20% of metastatic melanoma patients at the diagnosis of stage IV disease, and the CNS is a frequent initial site of disease progression for systemic therapies. Historically, patients with melanoma brain metastases (MBMs) had a dismal prognosis (median overall survival [OS] approximately 4 months).^2^ Fortunately, outcomes and our ability to estimate survival have improved.^3,4^ Nonetheless, the development of more effective strategies to predict, prevent and treat CNS metastases are critical to further improving survival in melanoma patients.

The majority of melanomas arise from the skin, and both epidemiological and molecular data strongly implicate ultraviolet radiation-induced DNA damage in the pathogenesis of this disease.^5^ Indeed, melanomas have more somatic mutations on average than any other common solid tumor type. While the majority of these mutations appear to be nonfunctional, most cutaneous melanomas harbor mutations that activate key oncogenic signaling pathways.^6^ This high prevalence of potentially targetable oncogenes and/or oncogenic signaling pathways has led to the testing and approval of many targeted therapies for this disease. While initial clinical trials excluded melanoma patients with active CNS disease, multiple studies subsequently demonstrated both the promise and the limitations of targeted therapies for these tumors. Notably, the high mutation burden that characterizes melanoma may also contribute to the high efficacy that has been seen with checkpoint inhibitor immunotherapies in this disease. However, concurrent treatment with steroids, which is frequently needed to control cerebral edema and/or symptoms from CNS metastases, has been shown to markedly diminish the efficacy of both single-agent and combination immunotherapy regimens, including in melanoma patients with brain metastases.^7,8^ Thus, the development of highly effective targeted therapy strategies remains an important goal in this disease, particularly for use in patients with CNS involvement.

## Molecular Biology of Melanoma

The Cancer Genome Atlas (TCGA) of cutaneous melanoma provides a robust picture of the molecular drivers of this disease (Table 1).^6^ Consistent with previous studies, the TCGA confirmed that activation of the RAS-RAF-MEK-ERK pathway is ubiquitous in this cancer. The RAS-RAF-MEK-ERK kinase signaling pathway was affected by mutations in more than 90% of the tumors in the TCGA, most commonly by point mutations in the *Braf* gene that encodes the serine-threonine protein kinase B-RAF. *Braf* mutations are detected in approximately 50% of cutaneous melanomas. More than 90% of these mutations result in substitutions for the valine at codon 600, most commonly to glutamic acid (approximately 70%; BRAF^V600E^) or lysine (approximately 20%; 90% BRAF^V600K^).^9^ These mutations increase the catalytic activity of BRAF by 100-200X and result in constitutive activation of the RAS-RAF-MEK-ERK pathway. A small (approximately 5%) subset of melanomas harbor mutations that affect other sites in the BRAF protein.^6^ These mutations have variable effects on the catalytic activity of BRAF, but virtually all seem to cause hyperactivation of downstream components in the RAS-RAF-MEK-ERK pathway.^10^ The pathway is also frequently activated by mutations in *Nras,* which were detected in 28% of the TCGA tumors.^6^ Mutations in *Nras* are mutually exclusive with hotspot (V600) mutations in *Braf* but they are detected in some tumors with mutations that affect sites in BRAF other than V600. Further, approximately 15% of cutaneous melanomas have loss of function mutations of *Nf1,* most (but not all) of which are nonoverlapping with the presence of hotspot mutations in *Braf* or *Nras*.^6^ As NF1 is a negative regulator of RAS proteins, these mutations again result in activation of RAS-RAF-MEK-ERK signaling. Finally, approximately 10% of the TCGA tumors harbored no mutations in *Braf*, *Nras*, or *Nf1*, and are termed “Triple Wild-Type” due to their lack of recurrent mutations in the RAF-RAF-MEK-ERK pathway.^11^ Interestingly, the Triple Wild-Type (WT) tumors had a low burden of point mutations overall, in contrast to most of the tumors with mutations in any of the other three genes. Additional analyses showed that the Triple WT cutaneous melanoma frequently harbor amplifications of oncogenes, perhaps providing additional therapeutic opportunities.^6^

**Table 1.** Targetable Recurrent Gene Alterations in Melanoma

Pathway	Gene	Type of Alteration	Frequency of Mutation	Possible Therapy Strategies	Agents
RAS-RAF-MEK-ERK pathway	BRAF 90% *Braf*^*V600*^ 10% *Braf*^*non-V600*^ NRAS NF1	Point mutation Mutation Mutation/loss of expression	~50% (CM) ~10-20% (AM) ~3-5% (MM) ~15-20% (CM) ~8-22% (AM) ~5-25% (MM) ~10-15%	BRAFi ± MEKi MEKi MEKi	Dabrafenib + trametinib Vemurafenib + cobimetinib Encorafenib + binimetinib Trametinib, cobimetinib, binimetinib Trametinib, cobimetinib, binimetinib
Cell cycle regulators	CDKN2A (p16^INK4A^ and P14^ARF^) CCND1 CDK4	Mutation/deletion Amplification Amplification/mutation	~20-40% ~11% ~15%	CDKi CDKi	Palbociclib (PD-0332991) Abemaciclib (LY2835210) Ribociclib (LEE011)
PI3K-AKT pathway	PIK3CA PTEN	Mutation Loss of function point mutations/deletions	~2.5% ~20-30%	Pan-PI3Ki Isoform-specific PI3Ki	Buparlisib (BKM120) Pictilisib (GDC-0941) Oncothyreon (PX-866) Alpelisib (BYL-719)
Receptor tyrosine kinases	KIT	Point mutation/amplification	<5% (without sun damage CM) 28% (chronically sun damaged CM) 3-36% (AM) 7-39% (MM)	KITi	Sunitinib, nilotinib, imatinib, sorafenib

AM, acral melanoma; CDKi, cyclin dependent kinases inhibitors; CM, cutaneous melanoma; MEK, mitogen-activated protein kinase inhibitors; MM, mucosal melanoma; PI3Ki, phosphoinositide 3-kinase inhibitors; UM, uveal melanoma.

The cutaneous melanoma TCGA also identified recurrent mutations in other clinically actionable pathways, including cell cycle regulators (affected in approximately 70%), the PI3K-AKT pathway (approximately 20%), and receptor tyrosine kinases (RTKs; approximately 1%; Table 1).^12^ Mutations in cell cycle genes most frequently included events (mutations, deletions, hyper-methylation) that result in loss of *Cdkn2a,* which encodes the cell cycle regulators P16 and P14, and which had previously been identified as a frequent germline event in cases of familial melanoma.^13^ Rare point mutations in *Cdk4,* and amplifications of *Cdk4* or *Ccnd1,* have also been detected in cutaneous melanomas. The PI3K-AKT pathway is activated rarely by hotpot mutations in the *Pik3ca*, which encodes the catalytic subunit of the lipid phosphatase PI3K, or in genes encoding the serine-threonine kinases *Akt1* or *Akt3.* The PI3K-AKT pathway can also be activated in melanoma by loss of expression or function of the tumor suppressor PTEN, which is a lipid phosphatase that counteracts PI3K and regulates this pathway. Loss of PTEN is detected in up to 20% of cutaneous melanomas, most commonly in tumors with a concurrent BRAF^V600^ mutation, thus resulting in concurrent activation of the RAS-RAF-MEK-ERK and PI3K-AKT signaling pathways.^14^ In addition to mutations in pathway components, the PI3K-AKT pathway can also be activated by alterations in growth factor receptors, particularly RTKs. Amplification of the genes encoding RTKs, including *Kit* and *Pdgfra,* are rare overall in cutaneous melanomas, but they are detected in up to 20% of the Triple WT subtype.^6^ Point mutations that activate the KIT RTK have also been detected as rare events in cutaneous melanomas, but they are detected in ≥ 20% of mucosal melanomas, which are rare but aggressive tumors that arise from mucosal surfaces throughout the body. Mucosal melanomas also have the same activating mutations in *Braf* and *Nras* that are detected in cutaneous melanomas, but at a lower prevalence (approximately 5% and 10%, respectively).^13^

Unexpectedly, molecular analyses of nevi (moles), benign lesions which very rarely give rise to invasive melanomas, showed that they also had a high rate of *Braf* and *Nras* mutations.^9^ This finding suggested that oncogenic mutations in the RAS-RAF-MEK-ERK pathway are very early events in the development and pathogenesis of cutaneous melanomas. This timing for these molecular events is consistent with the very high concordance that has been observed for activating mutations in the RAS-RAF-MEK-ERK signaling pathway between MBMs, extracranial metastases, and primary tumors.^15,16^ Indeed, this result is also consistent with the clinical activity that has been observed with FDA-approved BRAF inhibitors in metastatic melanoma patients with a BRAF^V600^ mutation and brain metastases, as will be described below. One study showed that brain metastases from multiple cancer types can feature mutations in key oncogenic signaling pathways, including in cell cycle regulators and the PI3K-AKT pathway, that are not easily detected in primary tumors.^17^ Another recent study revealed unique genetic variants that are exclusive in the MBMs but are absent in the corresponding extracranial metastases.^18^ However, currently available data has failed to find significant enrichment of private mutations in a specific gene or genes in MBMs.^16,19^

## Systemic Targeted Therapy in Melanoma

Historically, metastatic melanoma has been highly resistant to cytotoxic chemotherapies. Temozolomide, an alkylating chemotherapy agent, was frequently used historically in melanoma patients with brain metastases because it efficiently crosses the blood-brain-barrier (BBB). However, prospective clinical trials showed that temozolomide achieved clinical responses in ≤5% of melanoma patients with brain metastases.^2^ Thus, the discovery of recurrent mutations in *Braf* in approximately 50% of cutaneous melanoma patients presented the new opportunity to test targeted therapy strategies in this disease. Initial clinical trials with the nonselective BRAF inhibitor sorafenib produced dismal results, with clinical response rates of ≤5%.^9^ However, the treatment and outcomes of metastatic melanoma patients improved dramatically with the development of second-generation, mutation-specific BRAF inhibitors. In contrast to sorafenib, these drugs selectively inhibit the BRAF protein better than all other kinases and have increased affinity for BRAF proteins with a V600 mutation compared to the WT protein.^9^ This preferential recognition and inhibition resulted in improved activity and safety, leading to the modern era of targeted therapy for this disease.^20^

The first two mutant-selective BRAF inhibitors to be approved in patients with metastatic melanoma were vemurafenib (FDA approval in 2011) and dabrafenib (FDA approval in 2013).^20^ Both of these agents achieved rapid clinical responses and symptomatic improvement in patients with a BRAF^V600^ mutation in early-phase clinical trials. In these early trials, a small number of patients without a BRAF^V600^ mutation were included, none of whom responded. Unexpectedly, preclinical testing showed that both vemurafenib and dabrafenib caused hyperactivation of the RAS-RAF-MEK-ERK signaling pathway and increased tumor growth in melanomas without a BRAF^V600^ mutation, a phenomenon termed paradoxical pathway activation by BRAFi.^9^ Thus, subsequent clinical testing, and FDA approvals, of BRAFi were limited to melanoma patients with a BRAF^V600^ mutation. While vemurafenib and dabrafenib were developed independently, they demonstrated very similar clinical activity in phase III clinical trials. Both agents achieved RECIST-criteria clinical responses in approximately 50% of metastatic melanoma patients with a BRAF^V600^ mutation, and disease control in approximately 90% of patients. Both vemurafenib and dabrafenib demonstrated significant improvement in patient outcomes in phase III clinical trials versus standard of care chemotherapy, resulting in their regulatory approval.^9,20^

While both vemurafenib and dabrafenib significantly improved both response rates and progression-free survival (PFS) compared to standard chemotherapy, the duration of response (DOR) for single-agent BRAF inhibitor (BRAFi) therapy was relatively short. Both vemurafenib and dabrafenib achieved a median PFS of approximately 6 months, and approximately 90% of patients developed resistance and disease progression within a year of starting treatment. Investigations of both clinical specimens and preclinical models showed that the most common feature of tumors with acquired resistance was reactivation of the RAS-RAF-MEK-ERK signaling pathway.^21^ Interestingly, loss of the BRAF^V600^ mutation was not observed as a mechanism of resistance, nor were mutations in the coding region of the *Braf* gene. However, molecular analyses and functional studies showed that melanomas could become resistant to single-agent BRAFi treatment though the development of amplification of *Braf* and through mutations that produced splice variants of the BRAF protein. Activation of the RAS-RAF-MEK-ERK signaling pathway in resistant tumors can also be caused by activating mutations in *Nras*, *Mek1*, or *Mek2.* Notably, the finding of activating *Nras* mutations with concurrent *Braf* mutations in these post-BRAFi tumors contrasts with the mutual exclusivity seen between these mutations in treatment-naïve melanomas,^6^ and thus reflects the profound effect this targeted therapy has on disease biology. While these mechanisms were quite diverse, preclinical studies demonstrated that combining BRAFi targeted therapies with agents that blocked targets downstream in the RAS-RAF-MEK-ERK pathway could overcome the resistant phenotype that they caused.^21^ Unexpectedly, preclinical studies also suggested that combining BRAFi with MEK inhibitors (MEKi) could reduce the cutaneous toxicity frequently seen with single-agent BRAFi.^9^ These studies showed that the cutaneous toxicity of BRAFi was caused by paradoxical pathway activation of the RAS-RAF-MEK-ERK pathway in keratinocytes, which did not have BRAF^V600^ mutations. This activation was blocked by concurrent treatment with MEKi and suggested that combining BRAFi and MEKi could be beneficial not only for efficacy but also for tolerability.

Fortunately, these hypothesized benefits were borne out in multiple clinical trials. To date, three different BRAFi plus MEKi combination targeted therapy regimens have been approved for patients with a BRAF^V600^ mutation: dabrafenib and trametinib (DT; FDA approval, 2014), vemurafenib and cobimetinib (2015), and encorafenib and binimetinib (2018).^22–24^ Each of these combinations was approved based on phase III clinical trials that demonstrated improved clinical outcomes compared to treatment with single-agent BRAFi. All three regimens are highly active, with overall response rates (ORRs) of 70%-80%, disease control rates (DCRs) of 90%-95%, and median PFS of 11-15 months. To date, no clinical trials have compared the efficacy of these combination regimens against each other. In addition, all phase III trials of single-agent BRAFi, and of BRAFi and MEKi combination therapy, excluded melanoma patients that had active, untreated brain metastases.

To date, no other targeted therapies have been approved specifically for patients with metastatic melanoma. Clinical trials have assessed MEKi in patients with *Nras* mutations, including the phase III NEMO trial that compared binimetinib versus dacarbazine chemotherapy in patients.^25^ Binimetinib produced a better ORR (15% vs. 7%) and a small but statistically significant improvement in PFS (median 2.8 vs. 1.5 months, one-sided *P* < .001), but had no impact on OS (hazard ratio 1.00, *P* = .50). Thus, binimetinib was not approved for the treatment of metastatic melanoma patients with *Nras* mutations but it is included in National Comprehensive Cancer Network (NCCN) guidelines as an option for patients that have failed immunotherapy.^26^ A number of KIT inhibitors that are approved in other diseases with *Kit* mutations have also been evaluated in melanoma. Non-randomized trials of KIT inhibitors (ie, imatinib) in metastatic melanoma patients with a *Kit* mutation have reported response rates of approximately 10%-30%, with higher rates (up to 50%) reported for patients specifically with recurrent mutations seen in this disease (ie, L576P, K642E).^13^ Treatment with KIT inhibitors is recommended for metastatic melanoma patients with a *Kit* mutation who have progressed on or cannot tolerate immunotherapy.^26^

No clinical trials have been reported to date with single-agent MEK or KIT inhibitors in melanoma patients with brain metastases. However, both prospective and retrospective studies have evaluated both BRAFi with and without MEKi targeted therapy in melanoma patients with a BRAF^V600^ mutation with MBMs (Table 2).

**Table 2.** Completed Prospective Targeted Therapy Clinical Trials in Patients With Melanoma Brain Metastases

Study	Phase	Targeted Therapy	Number of Patients	Intracranial ORR (CR + PR)	Intracranial DCR (CR + PR + SD)	Intracranial PFS
Falchook et al.^27^	I	Dabrafenib	Cohort of untreated MBM (n = 10)	90%	—	4.2 months
Long et al.^28^ BREAK-MB	II	Dabrafenib	Cohort A: no previous local MBM therapy (n = 89) Cohort B: previous local MBM therapy (n = 83)	39.2% 30.8 %	81.1% 89.2%	16.1 months 16.6 months
McArthur et al.^29^	II	Vemurafenib	Cohort A: no previous local MBM therapy (n = 18) Cohort B: previous local MBM therapy (n = 56)	18% 18%	61% 59%	3.7 months 4 months
Davies et al.^32^ COMBI-MB	II	Dabrafenib + trametinib	Cohort A: *Braf*^V600E^ asymptomatic, with no previous local MBM therapy (n = 76) Cohort B: *Braf*^V600E^, asymptomatic, with previous local MBM therapy (n = 16) Cohort C: *Braf*^V600D/K/R^, symptomatic with or without local MBM therapy (n = 16) Cohort D: *Braf*^V600E/D/K/R^, symptomatic, with or without previous local MBM therapy (n = 17)	58% 56% 44% 59%	78% 88% 75% 82%	5.6 months 7.2 months 4.2 months 5.5 months

CR, complete response; DCR, disease control rate; MBM, melanoma brain metastases; ORR, overall response rate; PFS, progression-free survival; PR, partial response; SD, stable disease

### Single Agent BRAF Inhibitor Therapy for MBMs

The initial trials described above with both vemurafenib and dabrafenib excluded patients with active or untreated brain metastases at least in part due to concerns that these agents would not cross the intact BBB. In fact, this is sometimes thought to provide a safety advantage for new agents by reducing the risk of neurotoxicity. However, the BBB is frequently disrupted by the brain metastases, allowing intracranial penetration and activity even for agents that have suboptimal BBB penetration.^11^

Dabrafenib was evaluated early in its development in 10 metastatic melanoma patients with a BRAF^V600^ mutation and asymptomatic brain metastasis, and nine patients showed evidence of intracranial response.^27^ Subsequently, the BREAK-MB phase II trial investigated the safety and efficacy of dabrafenib as monotherapy in 172 patients with metastatic melanoma with a BRAF^V600^ mutation and untreated or progressing brain metastasis(es).^28^ While the trial was not randomized, the patients were categorized as Cohort A (n = 89; no previous local treatments to the brain) and Cohort B (n = 83; previous brain-directed treatments, including surgery and/or radiation) for separate analyses. Separate analyses were also performed to evaluate outcomes in patients with a BRAF^V600E^ mutation (n = 74 in Cohort A; *n* = 65 in Cohort B) or a BRAF^V600K^ mutation (n = 15 in Cohort A; n = 18 in Cohort B). BREAK-MB showed that dabrafenib was safe in patients with brain metastases, with no new or unexpected toxicities observed. Consistent with the earlier preliminary results, BREAK-MB showed that dabrafenib has significant activity in MBMs. For patients with a BRAF^V600E^ mutation, the intracranial ORR (iORR) was 39% and the intracranial DCR (iDCR) was 81% for Cohort A, and 31% and 89.2%, respectively, for Cohort B. Interestingly, lower activity was observed in patients with a BRAF^V600K^ mutation (ie, iDCR 33% in Cohort A, 50% in Cohort B). The median intracranial DOR (iDOR) and OS for patients with a BRAF^V600E^ mutation were 5 and 8.25 months in Cohort A, and 7 and 7.75 months in Cohort B.

A phase II study investigated the safety and efficacy of single-agent vemurafenib in 146 patients with a *Braf*^*V600*^ mutation and active brain metastases.^29^ The outcomes were again analyzed separately for patients with untreated (Cohort 1, n = 90) and previously treated (Cohort 2, n = 56) brain metastases. The outcomes were relatively similar in both cohorts. In Cohort 1, the OIRR was 29%, the median PFS was 3.7 months, and the median OS was 8.9 months. In Cohort 2, the iORR was 23%, the median PFS was 4 months, and the median OS was 9.6 months. Similar to patients with extracranial disease, no randomized trials compared dabrafenib and vemurafenib, but preclinical studies suggest that dabrafenib may achieve better intracranial delivery.^30,31^

### BRAF + MEK Inhibitor Combination Therapy in MBMs

The first trial to investigate the safety and efficacy of the combined BRAF and MEK inhibition in patients with BRAF^V600^-mutant melanoma patients with MBMs was COMBI-MB. All patients in the trial (n = 125) were treated with the FDA-approved dosing regimen of DT.^32^ The trial again included multiple cohorts that all received the same treatment. The largest group, Cohort A (n = 76), included patients with a BRAF^V600E^ mutation and no previous brain-directed treatments. Brain metastases had to be asymptomatic, but patients could be on a stable or decreasing dose of steroids to achieve this. The other cohorts in the trial were all small and thus exploratory. Cohort B (n = 16) included patients with a BRAF^V600E^ mutation, asymptomatic brain metastasis, and prior brain-directed therapy. Cohort C included patients with a BRAF^V600K/D/R^ mutation and asymptomatic brain metastasis, with or without previous brain-directed therapy. Cohort D (n = 17) included patients with symptomatic brain metastases (any BRAF^V600^ mutation, with or without prior treatment to the brain). The trial excluded patients with leptomeningeal disease (LMD) or MBMs > 4 cm in diameter. In Cohort A, the iORR was 58% and the iDCR was 78%, similar to the response rates observed in extracranial melanoma metastases (extracranial ORR 55%, extracranial DCR 79%). Despite their clinical differences, the outcomes in the other three cohorts were similar, and no new or unexpected toxicities with DT treatment were reported. While the safety and initial response rates were promising the DORs were relatively short. The median intracranial PFS in Cohort A was 5.6 months, which was shorter than the duration of control of extracranial metastases in this cohort (10.2 months), and less that had been observed in pooled analyses of DT in trials in patients without CNS involvement (approximately 11 months).^33^

There is currently limited data regarding the efficacy of the other two approved BRAFi + MEKi combinations in MBM patients. A retrospective study of MBM patients (n = 24) treated with encorafenib and binimetinib reported an iORR of 33%.^34^ While this appears to be lower than the response rate for DT, most (n = 21) of the patients had previously been treated with BRAFi with or without MEKi. While the fact that patients did respond is consistent with prior studies showing that resistance to inhibitors of the RAS-RAF-MEK-ERK pathway is not necessarily permanent, and supports the rationale for re-challenge in patients, it makes comparison to the results of COMBI-MB challenging.^35^ Encorafenib and binimetinib are currently being evaluated in a phase II study in MBM patients with a BRAF^V600^ mutation (NCT03911869; Table 3). To date, no prospective data has been reported for vemurafenib and cobimetinib in MBM patients.

**Table 3.** Ongoing Trials of Targeted Therapy in Patients With Melanoma Brain Metastases

Therapy(ies)	Name of Study	NCT
Targeted therapy	Vemurafenib Plus Cobimetinib Combination in BRAF Mutated Melanoma with Brain Metastases (CONVERCE)	NCT02537600
	An Open-Label, Randomized, Multicenter Trial of Encorafenib + Binimetinib Evaluating a Standard-dose and a High-dose Regimen in Patients with BRAFV600-mutant Melanoma Brain Metastasis (POLARIS)	NCT03911869
	A Phase 2 Study of Abemaciclib in Patients with Brain Metastases Secondary to Hormone Receptor Positive Breast Cancer, Non-small Cell Lung Cancer, or Melanoma	NCT02308020
	Buparlisib in Melanoma Patients Suffering from Brain Metastases (BUMPER)	NCT02452294
	Safety of ABM-1310 Monotherapy in Patients with Advanced Solid Tumors	NCT04190628
	A FIH Study of PF-07284890 in Participants with BRAF V600 Mutant Solid Tumors with and without Brain Involvement	NCT04543188
	E6201 Plus Dabrafenib for the Treatment of Metastatic Melanoma Central Nervous System Metastases	NCT03332589
	Genetic Testing in Guiding Treatment for Patients with Brain Metastases	NCT03994796
Targeted therapy and radiotherapy	Concurrent Trametinib + Dabrafenib with Stereotactic Radiation in BRAF Mutation- Positive Malignant Melanoma and Brain Metastases	NCT02974803
	A Phase II Study of Vemurafenib Plus Cobimetinib After Radiosurgery in Patients With BRAF-mutant Melanoma Brain Metastases (RadioCoBRIM)	NCT03430947
	Anti-PD 1 Brain Collaboration + Radiotherapy Extension (ABC-X Study)	NCT03340129
Targeted therapy and immunotherapy	A Study to Compare the Administration of Encorafenib + Binimetinib + Nivolumab versus Ipilimumab + Nivolumab in BRAF-V600 Mutant Melanoma with Brain Metastases	NCT04511013
	A Study Evaluating the Safety and Efficacy of Cobimetinib Plus Atezolizumab in BRAFV600 Wild-type Melanoma with Central Nervous System Metastases and Cobimetinib Plus Atezolizumab and Vemurafenib in BRAFV600 Mutation-positive Melanoma with Central Nervous System Metastases	NCT03625141
	Nivolumab With Trametinib and Dabrafenib, or Encorafenib and Binimetinib in Treating Patients with BRAF Mutated Metastatic or Unresectable Stage III-IV Melanoma (Patients with melanoma brain metastases are allowed)	NCT02910700

## Combinatorial Approaches for MBMS

### Targeted therapy and Radiotherapy

There is a strong rationale to combine targeted therapies and radiation in MBMs. First, as local control with stereotactic radiosurgery (SRS) is better for smaller than larger tumors, the ability of combined BRAF and MEK inhibition to reduce tumor size in most patients may augment the efficacy of SRS. In addition*, Braf* and *Nras* mutations were associated with worse outcomes with SRS in retrospective studies, thus inhibiting the RAS-RAF-MEK-ERK pathway may be beneficial.^36^ In turn, radiation can disrupt the BBB and thereby allow better penetration of targeted therapies into MBMs. In addition to these theoretical advantages, preclinical studies support the potential for synergy when radiation is combined with BRAF inhibition, and retrospective reports suggest improved outcomes in patients treated with both modalities compared to historical controls.^37–39^ A phase II study (NCT01721603) was designed to evaluate the combination of dabrafenib with SRS for safety and 6-month distant brain metastasis–free survival rate in MBM patients, but it was closed due to low accrual. RadioCoBRIM, an ongoing phase II open label study (NCT03430947), is testing the safety and activity of vemurafenib plus cobimetinib after radiosurgery in MBM patients (Table 3).

Although several retrospective studies have examined outcomes with SRS used in combination with targeted therapies,^40–42^ clinical recommendations remain limited in the absence of robust prospective data. More prospective trials are needed to address strategies to optimize timing and sequencing of combination therapy to maximize synergy with minimal or acceptable toxicity. Potential toxicities for combinations of radiation and targeted therapy include rash, hemorrhage, headache, cerebral edema, and radiation necrosis.^43–45^ Consensus guidelines from the Eastern Cooperative Oncology Group have recommended holding BRAF inhibitors for at least one day before and after SRS and for at least three days before and after fractionated radiation therapy.^46^ However, there are currently no guidelines for BRAF + MEK inhibitor combination regimens, which have much less cutaneous toxicity than single-agent BRAF inhibitor treatment.

### Targeted Therapy and Immunotherapy

Multiple studies have shown that BRAF ± MEK inhibition can reverse the immunosuppressive effects of the BRAF^V600^ mutation, supporting the rationale to combine these targeted therapies with checkpoint inhibitor immunotherapy.^47^ In 2020, the FDA approved the first such regimen, vemurafenib + cobimetinib + atezolizumab (ant-PD-L1 antibody), based on the results of the IMspire150 phase III trial.^48^ The addition of atezolizumab did not improve the response rate achieved with targeted therapy alone, but it increased the duration of the responses. Thus, perhaps the addition of immunotherapy could address the short duration of intracranial responses observed with DT in the COMBI-MB trial.^32^

Currently, several combinations of immunotherapy and targeted therapy are being tested in MBM. The activity and safety of nivolumab combined with DT is being evaluated in a phase II study in BRAF-mutant patients, including patients with MBMs (NCT02910700). The activity and safety of atezolizumab combined with vemurafenib and cobimetinib in MBMs is being evaluated in another phase II study (NCT03625141).

### Other Targets in MBMs

Loss of the PTEN tumor suppressor, which activates the PI3K-AKT pathway, was initially discovered as a highly recurrent event in primary brain tumors (GBMs).^14^ Interestingly, there is now growing data implicating activation of the PI3K-AKT pathway in brain metastases from melanoma and other tumor types.^49^ Multiple studies have identified increased activation of the PI3K-AKT pathway in MBMs, including in comparison to extracranial metastases from the same patients.^16,50^ It appears that this molecular phenotype could be caused by multiple mechanisms. Loss of PTEN in is associated with increased risk of developing brain metastases in stage III melanoma patients and increases the rate of brain metastasis formation in a mouse model of BRAF-mutant melanoma.^51,52^ Although less common than PTEN loss, the PI3K/AKT pathway can be hyperactivated by mutation of E17K in AKT isoforms. One study showed that mice harboring melanoma tumors expressing AKT1^E17K^ had the highest risk for brain metastasis, an effect mediated by FAK, and thus supporting the rationale for the therapeutic targeting of AKT and/or FAK.^53^ The pathway may also be activated as a late of selective event in the brain. Two independent proteomic analyses of resected MBMs and extracranial metastases from individual patients and identified increased activation of the PI3K-AKT pathway, but not the RAS-RAF- MAPK pathway, in each of the brain metastases.^16,50^ While these results could be explained by clonal selection as cells with pathway activation to form brain metastases, there is also evidence that the PI3K-AKT pathway can be activated by the brain microenvironment. One study showed that astrocyte secrete exosomes containing miRNAs that target PTEN, resulting in decreased PTEN expression and increased PI3K-AKT pathway activation in both melanoma and breast cancer brain metastases.^54^ Another study showed that cerebrospinal fluid activates the PI3K-AKT pathway in human melanoma cells.^31^ Importantly, activation of the PI3K-AKT pathway, and/or loss of PTEN, has been implicated in resistance in to both targeted therapy and immunotherapy in melanoma patients and preclinical models.^14,49^ Further, preclinical studies have shown that PI3K-AKT inhibitors can have activity against MBMs, including in combination with BRAF inhibitors.^16,55^ Interestingly, a recent study using in vivo molecular imaging by intravital multiphoton microscopy further investigated the temporo-spatial dynamics of PI3K-AKT pathway activation during brain metastasis formation. The study implicated the PI3K-AKT pathway as a critical requirement early in colonization of brain tissue by cancer cells. Further, there studies showed that inhibition of the PI3K-AKT pathway effectively prevented MBM formation.^56^

While these studies strongly implicate the PI3K-AKT pathway in MBMs, the clinical development of PI3K-AKT inhibitors has been challenging due to the toxicities incurred by these agents, and currently there are no ongoing studies evaluating inhibitors of this pathway in MBMs patients. Notably, recent studies suggest that increased activation of the PI3K-AKT pathway in brain metastases compared to patient-matched primary tumors and extracranial metastases is observed in many different tumor types, further supporting the rationale for therapeutic development and clinical trials in this area.^17,57^

Whole genomic profiling studies have recently implicated metabolic pathways as a novel feature and therapeutic target in brain metastases. RNA sequencing of patient-matched brain and extracranial metastases from melanoma patients unexpectedly identified increased expression of genes involved in oxidative phosphorylation (OXPHOS) in MBMs.^19^ This finding was recapitulated in a murine model in which melanoma primary tumors give rise to both lung and brain metastases. Increased OXPHOS was also confirmed by direct metabolite analysis and in vivo metabolic tracing of MBMs established in mice by direct intracranial injection. Interestingly, while each of these melanoma models harbored a BRAF mutation, single-agent treatment with an experimental direct OXPHOS inhibitor, IACS-010759, significantly improved the survival of mice- and had greater effects on brain metastases than on lung metastases or primary tumors. A follow-up study showed that the increased expression of OXPHOS genes, and increased sensitivity to OXPHOS inhibition, was also seen in brain metastases from lung cancer, breast cancer, and renal cell carcinoma.^57^ Recently, investigators characterizing cancer cells with increased metastatic potential to the brain implicated an additional metabolic dependency of brain metastases, glucose-derived serine biosynthesis. These studies showed that the brain microenvironment has very low levels of serine and glycine, which thus must be synthesized by cancer cells to survive in the CNS.^58^ Thus, the brain-metastatic cancer cells markedly upregulated 3-phosphoglyerate dehydrogenase, the rate-limiting enzyme in glucose-derived serine synthesis, to achieve this. In addition to confirming this adaptation in brain metastases from patients with melanoma, breast cancer, and lung cancer, they showed that inhibitors of this pathway inhibited the growth of brain metastases in mice but not tumors growing at other sites, similar to the brain-preferential activity observed with OXPHOS inhibition.

## Conclusion

Targeted therapies can achieve tremendous clinical benefit in MBM patients with activating mutations in the *Braf* gene. Indeed, the clinical activity and safety seen with BRAF ± MEK inhibitors in post-approval trials in MBM patients raises questions about why patients with MBMs continue to frequently be excluded from clinical trials. In fact, there is now widespread support for widening eligibility for clinical trials to include selected patients with brain metastases, including guidance from the FDA^1^ and recommendations from ASCO.^4,59^ Excluding such patients is not only discrimination but slows trial accrual clinical progress. Removing such barriers will almost certainly result in improved outcomes for MBM patients by providing access to more treatment options. Addressing several key questions from existing studies will further improve the impact of targeted therapy for MBMs.

The COMBI-MB study demonstrated that combined treatment with DT achieved high rates of intracranial response and DCRs in patients with MBMs. However, the duration of the responses in brain metastases was much shorter than in extracranial metastases in the patients in the trial, and compared to prior clinical trials in patients without CNS involvement.^32,33^ It is currently unknown why the DOR in MBMs was shorter- and thus what is the best way to improve upon these results. Previous studies with BRAF inhibitors (alone and in combination with MEK inhibitors) have shown that deeper inhibition of the RAS-RAF-MEK-ERK pathways correlates with better clinical outcomes in patients with extracranial metastases.^9^ If incomplete pathway inhibition is being achieved in MBMs, then outcomes could be improved with increased drug delivery to the CNS by higher dosing of current agents (ie, NCT03911869). Alternatively, perhaps outcomes could be improved through the use of BRAF and MEK inhibitors designed to penetrate the BBB more efficiently, a strategy currently being evaluated in several ongoing clinical trials inhibitors with improved BBB penetration (ie, NCT03332589). A second possibility is that the shorter DOR in the brain metastases is due to the decreased immune infiltration of these tumors,^19,49^ which has also been shown to correlate with inferior outcomes with BRAF and MEK inhibitors.^60^ As noted above, recent trials have shown that the addition of checkpoint inhibition to BRAF and MEK inhibitors improved the duration of clinical responses in patients with extracranial disease only,^48^ so the results of an ongoing trial of triplet therapy in MBM patients are eagerly awaited. Alternatively, studies of MBMs have identified that these tumors harbor increased activation of the PI3K-AKT pathway and OXPHOS, both of which have previously been shown to cause resistance to BRAF and MEK inhibitors.^14,61,62^ Preclinical studies with PI3K-AKT pathway inhibitors, including in MBM models, suggest that they may have positive anti-tumor effects in combination with BRAF and MEK inhibitors,^16,55^ but this has yet to be evaluated in patients with brain metastases. However, such combinations will need to have acceptable toxicity profiles, particularly to allow for maintenance of dosing that achieves target inhibition in the brain, which has been quite challenging for PI3K-AKT pathway inhibitors alone and in combination with other agents. Finally, in addition to other systemic therapies, available data suggests that combining BRAF and MEK inhibitors with stereotactic radiation may have increased efficacy, a strategy that is currently being evaluated in prospective clinical trials.

There are many targetable oncogenes in melanoma beyond *Braf.*^6^ While KIT inhibitors have been evaluated in multiple clinical trials in melanoma patients with *Kit* mutations, there is no data currently available regarding the activity of these agents in MBM patients. Similarly, while the MEK inhibitor binimetinib is part of NCCN recommendations for metastatic melanoma patients with *Nras* mutations, there is also no data reported to date about single-agent MEK inhibitors in MBM patients. Alterations in *Cdkn2a* and other cell cycle regulators are almost universal in cutaneous melanomas, and they may frequently have additional mutations detected in brain metastases.^6,17^ Thus, inhibitors against components of this pathway (ie, CDK4/6 inhibitors) may also be rational to evaluate in MBMs (ie, NCT02308020).

In addition to cell signaling pathways, recent studies have also identified unique metabolic features and dependencies of MBMs, including OXPHOS and glucose-derived serine synthesis.^19,57,58^ Intriguingly, in each of these studies metabolic inhibitors showed better anti-tumor activity against brain metastases compared to primary tumors or tumors growing at other metastatic sites. Notably, this preferential activity against brain metastases suggests that inhibitors of these metabolic pathways will need to be combined with other agents, as most patients with MBMs have concurrent extracranial disease.^1^ Similar to the combinations discussed above such evaluations will need to carefully assess safety in addition to efficacy and should include not only systemic therapies but also radiation.^63^ While such studies remain to be done to optimize translational strategies, the results also suggest a new paradigm for clinical testing. While generally most agents have been evaluated in brain metastases only after initial demonstrating efficacy in patients without CNS involvement, this strategy has the risk of eliminating agents that may have their greatest impact on brain metastases. Thus, further improvements in the efficacy of targeted therapy for MBMs will required both laboratory and clinical investigators to challenges paradigms and focus specifically on the unique challenges they present.

## Footnotes

1
https://www.fda.gov/media/121317/download.
